# Interaction of the Gut-Liver-Brain Axis and the sterolbiome with sexual dysfunction in patients with cirrhosis

**DOI:** 10.1080/19490976.2024.2446390

**Published:** 2025-01-07

**Authors:** Spencer C. Harris, Jasmohan S. Bajaj

**Affiliations:** Division of Gastroenterology, Hepatology, and Nutrition, Virginia Commonwealth University and Richmond VA Medical Center, Richmond, VA, USA

**Keywords:** sex hormone, gut-liver-brain axis, alcohol, obesity, fecal microbiota transplant, hepatic encephalopathy

## Abstract

There is a complex interplay between the gut microbes, liver, and central nervous system, a gut-liver-brain axis, where the brain impacts intestinal and hepatic function while the gut and liver can impact cognition and mental status. Dysregulation of this axis can be seen in numerous diseases. Hepatic encephalopathy, a consequence of cirrhosis, is perhaps the best studied perturbation of this system. However, patients with cirrhosis have been shown to have increased incidence of other disorders of mental health which may be otherwise less clinically identifiable. Sexual dysfunction affects a large proportion of patients with cirrhosis and is associated with decreased quality of life. Screening for sexual dysfunction in patients with cirrhosis is often overlooked, and even when identified, treatment options are limited, particularly in patients with advanced liver disease. The mechanism by which patients with cirrhosis develop sexual dysfunction is multifactorial, but a key driver of this clinical manifestation is alterations in circulating sex hormones. In patients with cirrhosis, low serum sex hormones have been shown to be associated with higher mortality regardless of MELD score. The gut microbiome has been shown to have an immense metabolic capacity to metabolize steroid hormones. This “*sterolbiome*” has already been implicated in other disease processes and has been linked to low circulating sex hormones, suggesting a new mechanism by which sex hormones may be altered in disease states where the gut-liver-brain axis is disrupted. The aim of this review is to cover sex hormone changes and sexual dysfunction in cirrhosis, examine the gut microbiome and its metabolic capacity, particularly for steroid hormones, and consider how microbial changes using fecal microbiota transplant could modulate sexual dysfunction.

## Introduction

The dysregulation of the gut-liver-brain axis that occurs in chronic liver disease can lead to major clinical consequences. Hepatic encephalopathy (HE) is a well-established consequence of gut-liver-brain axis dysregulation, and its multi-factorial causes underline the complex mechanisms by which these systems are interconnected. Impaired daily function, which occurs independent of HE, is a major determinant of sexual dysfunction, remains largely underrecognized in patients with cirrhosis.

Sexual dysfunction is a broad term encompassing several aspects of sexual health. These include dysfunctions of sexual desire, dysfunction of sexual arousal, orgasmic dysfunction, dyspareunia, and sexual aversion.^1^ Epidemiologic studies estimate that the prevalence of sexual dysfunction reaches upward of 30% of men and 45% of women.^1^ Common risk factors associated with sexual dysfunction for men and women include changes in circulating sex hormones that occur in chronic diseases, including cirrhosis. Cirrhosis is characterized by alterations in circulating sex hormones, and sexual dysfunction in this population is underdiagnosed and undertreated.

Commensal gut microbes have previously been shown to be capable of impacting the levels of circulating sex hormones.^2^ Alterations in circulating sex hormones, such as the normal physiological changes that occur during pregnancy, have also been noted to impact the composition of the gut microbiome.^3^ Changes to the gut microbiome have previously been linked to numerous mental illnesses, including depression and anxiety.^4^ Studies in mouse models have shown that changes in the gut microbiome occur in chronically stressed mice, and that corrections of these perturbations can ameliorate these behavioral changes.^5^ Chronic liver disease is associated with perturbations of the gut microbiome, dysregulated serum sex hormones, and high rates of sexual dysfunction. Therapeutic targeting of the microbiome to alleviate these dysfunctions warrants further investigation.

## What happens to sex hormones in cirrhosis?

Cirrhosis is a disease state characterized by “feminization”, consisting of low serum testosterone and high serum estrogen. Serum testosterone is decreased in up to 90% of patients with cirrhosis, and the decrease in serum testosterone is more profound as liver disease severity increases.^6^ Women with chronic alcohol use disorder have been shown to have elevated levels of prolactin and androstenedione when compared to healthy controls, which has been attributed to sexual hormone changes found in female patients with more advanced liver disease.^7^

The mechanism by which sex hormones are altered in cirrhosis is multifactorial. A healthy liver plays a crucial role in the synthesis of many proteins, including sex hormone-binding globulin (SHBG) and albumin, which are responsible under normal physiological conditions to bind testosterone in the blood stream leading to decreased androgenic effects.^8^ Studies have shown that SHBG levels are commonly elevated in cirrhosis.^9^ Although seemingly counterintuitive in the setting of cirrhosis, low serum testosterone can lead to an increase in hepatic production of SHBG, leading to an increase in the binding of remaining testosterone. This leads to further decrease in circulating testosterone levels and decreased serum androgenic activity of testosterone. Additionally, the shunting of blood flow away from the liver to peripheral tissue is thought to contribute to decreased testosterone. Increased peripheral tissue aromatization, which converts testosterone into estrogen, further exacerbates the “feminization” state in cirrhosis.^10^ However, this conversion of androgens to estrogens is not the only means by which sex hormone levels are altered in cirrhosis.

In addition to changes in the circulating levels of testosterone, studies have shown significant changes in serum estrogen and its metabolites in patients with cirrhosis. The three major estrogens are estrone (E1), estradiol (E2), and estriol (E3), of which E2 has the most estrogenic activity when bound to the estrogen receptor.^11^ Men with cirrhosis have been shown to have elevated serum E2 levels, along with elevated E2:testosterone ratios.^12^ The peripheral conversion of androgens to estrogen compounds due to portal hypertension is thought to be one mechanism by which these ratios are altered. Additionally, the liver is responsible for glucuronidation of estrogen compounds to facilitate their excretion.^13^ However, glucuronidation is decreased in patients with cirrhosis, leading to further increases in serum estrogen levels.^9^

Cirrhosis is also associated with central hypogonadism. Gonadotropin-releasing hormone (GnRH) is released from the hypothalamus, acting on the anterior pituitary gland. When stimulated, the anterior pituitary gland releases both luteinizing hormone (LH) and follicle stimulating hormone (FSH), which under normal physiological conditions act on the gonads to stimulate production of androgenic compounds. Pituitary produced LH is also inappropriately normal or suppressed in the setting of low circulating testosterone levels.^9^

There are major clinical sequelae that patients with cirrhosis suffer from due to low serum testosterone. Common clinical manifestations of low testosterone include weakness, fatigue, and depression.^9^ A recent meta-analysis of 20,654 individuals showed that low testosterone and elevated SHBG are associated with all-cause mortality.^14^ Studies of men with cirrhosis have shown an association between mortality and low serum testosterone independent of MELD score.^15,16^ Sarcopenia, an independent predictor of mortality in cirrhosis, is exacerbated by low testosterone. A retrospective analysis showed that males with cirrhosis and sarcopenia had significantly lower testosterone levels than males with cirrhosis without sarcopenia.^17^ A trial of testosterone replacement therapy in males with cirrhosis showed significant improvement in muscle and bone mass, muscle function, and a reduction in hemoglobin A1c, with a trend toward reduction in all-cause mortality.^18^ The changes in circulating sexual hormones and disruptions in the hypothalamic-pituitary-gonadal axis that occur in cirrhosis can also lead to sexual dysfunction.

## Sexual dysfunction in cirrhosis: a multifactorial problem

**Figure 1.**
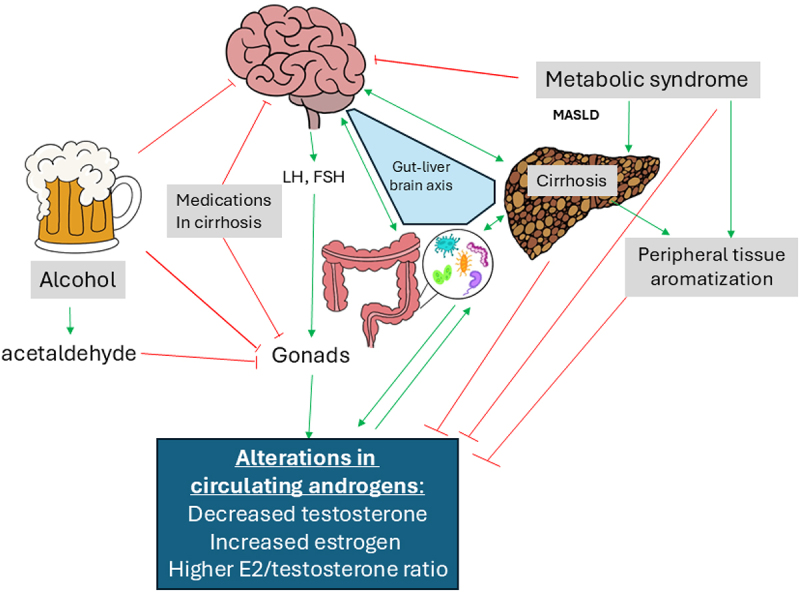
Schematic showing the multi-factorial influences on circulating androgens in patients with chronic liver disease and cirrhosis.

Sexual dysfunction, a broad term encompassing libido, fertility, and erectile dysfunction/vaginal lubrication, affects a significant proportion of patients with cirrhosis. Prevalence rates above 50% have been reported in numerous studies.^19,20^ Prior studies have shown a strong association between sexual dysfunction and overall quality of life in patients with chronic liver disease.^21^ The mechanism by which patients with cirrhosis develop sexual dysfunction is multifactorial (Figure 1). The etiology of liver disease, pathophysiologic changes in liver disease, and the medical treatments for the consequences of cirrhosis have all been implicated as potential contributors to sexual dysfunction.

In the United States, alcohol is the leading cause of chronic liver disease. Prior studies in animal models have shown that testosterone levels are suppressed and that LH and FSH levels are inappropriately normal in both acute and chronic alcohol exposure.^22^ Ethanol, along with its metabolite acetaldehyde, has a directly toxic effect on gonadal cells via oxidative injury, which can lead to primary testicular dysfunction.^23–25^ Ethanol also decreases nitric oxide, contributing to erectile dysfunction in males with cirrhosis.^26^

Metabolic-associated fatty liver disease and associated metabolic syndrome have separate mechanisms by which they can cause sexual dysfunction. Obesity is associated with secondary hypogonadism.^27^ Obesity also leads to insulin resistance, which is associated with a decrease in sex hormone-binding globulin production which over time suppresses the overall production of sex hormones.^27^ Excess adipose tissue, particularly visceral fat, causes aromatization of testosterone into estradiol, decreasing available testosterone and suppressing the release of gonadotropin releasing hormone and luteinizing hormone.^28^ Chronic low-grade systemic inflammation associated with obesity leads to increased circulating inflammatory cytokines, which can disrupt the HPG axis leading to decreased sex hormone production.^27^ Studies have shown that patients with MASH have higher rates of low-free testosterone.^29^ Additionally, men with MASLD and low-free testosterone were more likely to have MASH and advanced fibrosis.^30^ Low testosterone and low sex hormone-binding globulin are both independent predictors of metabolic syndrome, a risk factor for MASH and MASLD.^31^

The treatments for the pathologic sequelae of cirrhosis have also been implicated as causes of sexual dysfunction in cirrhosis. Nonselective beta blockers are used as primary or secondary prophylaxis to prevent variceal bleeding. Use of these medications has also been associated with sexual dysfunction due to decreased arousal in both men and women.^32^ Spironolactone, typically used alongside furosemide in cirrhosis in the medical treatment of ascites, has anti-androgenic properties which can lead to decreased libido and erectile dysfunction by blocking the androgen receptor.^33^

Evaluation of sexual dysfunction can be difficult in the clinical environment and is therefore often overlooked in this patient population. There are numerous different assessments for sexual dysfunction. The Arizona Sexual Experience Scale (ASEX) is a five-item rating scale and was developed to broadly evaluate areas contributing to sexual dysfunction including drive, arousal, penile erection, ability to reach orgasm, and satisfaction.^34^ ASEX has been well validated in numerous studies on sexual dysfunction.^34^ Sex-specific questionnaires can also be used to assess sexual dysfunction. The Female Sexual Function Index (FSFI) was designed to assess sexual function across numerous domains including desire, arousal, lubrication, orgasm, satisfaction, and pain.^35^ The International Index of Erectile Function (IIEF) is also well studied in the evaluation of male sexual dysfunction and queries numerous domains including erectile function, orgasmic function, sexual desire, intercourse satisfaction, and overall satisfaction.^36^ It is important to note that these surveys evaluating sexual dysfunction were developed on and validated in cis-heterosexual populations, and their effective use in screening has not been well studied in LGBTQIA+ populations.^37^

The treatment of sexual dysfunction with first-line medical therapies, such as phosphodiesterase-5 (PDE-5) inhibitors, is typically avoided in patients with cirrhosis due to their potential side effects to reduce mean arterial pressure and the need for dose adjustment in advanced liver disease.^38^ There was a recent clinical trial that showed PDE-5 inhibitor tadalafil improved symptoms of sexual dysfunction and improved quality of life in patients with compensated cirrhosis.^39^ However, there is insufficient evidence in decompensated cirrhosis regarding safety of the PDE-5 inhibitors given their blood pressure lowering adverse effects. Potential medical therapies for sexual dysfunction in females with cirrhosis are similarly limited, although studies have shown improvement in sexual dysfunction in females with cirrhosis after liver transplantation, suggesting a critical role cirrhosis plays in sexual dysfunction in this population as well.^40^

Sexual dysfunction in patients with cirrhosis is a critical aspect of overall quality of life, is underrecognized in clinical encounters, and is undertreated due to concern for medication side effects. However, even as we have a better understanding of the factors in the host that impact the physiology of sexual dysfunction in patients with cirrhosis, new evidence suggests a substantial capacity for the gut microbiome both in health and disease to modulate host sexual hormone levels, which may represent an underappreciated avenue by which patients with cirrhosis develop sexual dysfunction.

## Sterolbiome: an essential component of gut microbiome metabolic potential

To understand how the gut microbiome may play a role in sexual dysfunction in patients with cirrhosis, it is important to review its published propensity to metabolize host sex steroid hormones. As with any niche in a symbiotic ecosystem, there is an evolutionary opening that permits abundant but otherwise unused nutrients to be utilized. Sharing the same four cycloalkane rings as bile acids, endogenously produced steroid hormones are conjugated to glucuronic or sulfuric acid and then excreted into bile.^41^

**Figure 2.**
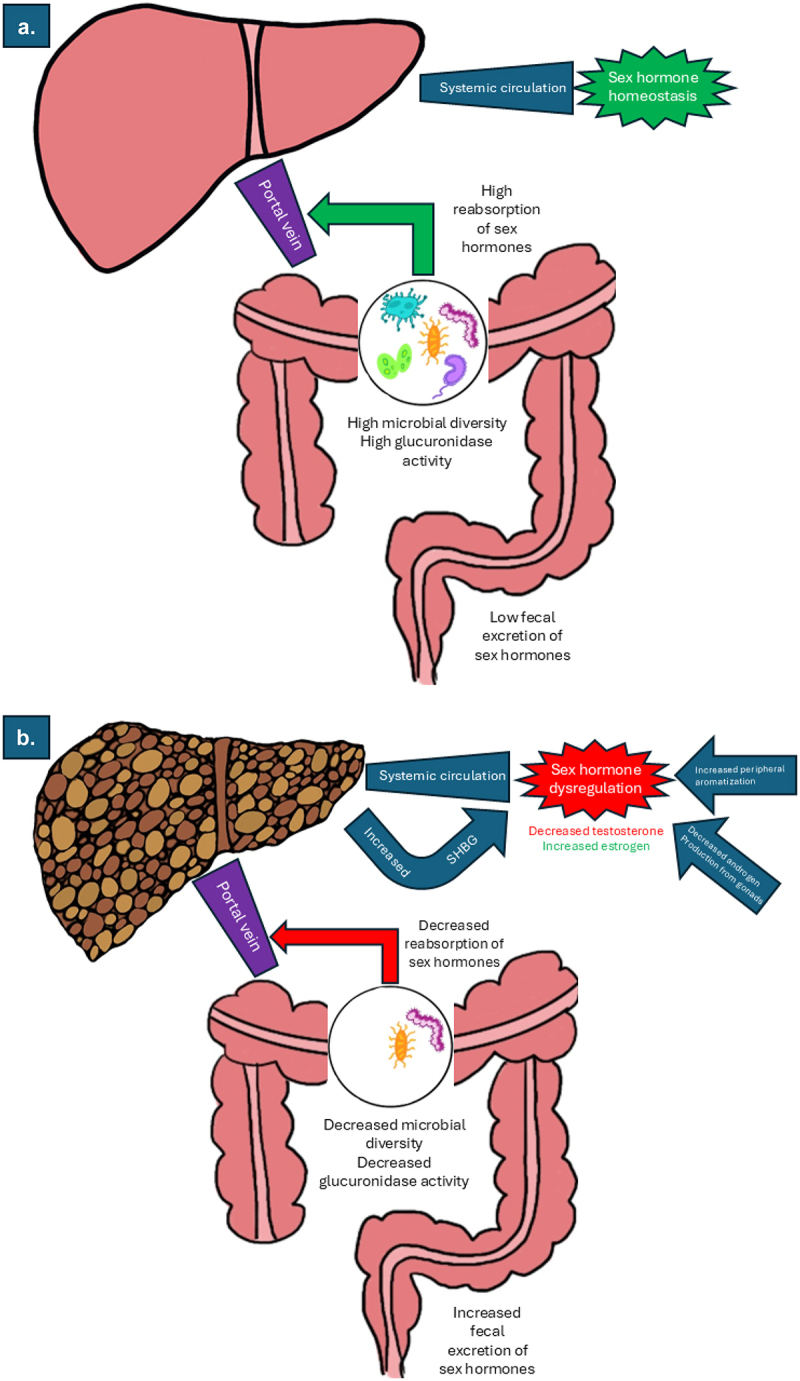
Changes in gut microbiome and circulating sex hormones in health (2a) and in cirrhosis (2b).

Inside the lumen of the intestines, conjugated sex steroid hormones can be deconjugated via hydrolysis, which liberates the free sex hormones to be reabsorbed or to undergo further metabolism by the gut microbiome, which is disrupted in cirrhosis (Figure 2a,b). Glucuronidase activity is found in some of the most abundant microbes in the gut microbiome, such as many *Bacteroides* species.^42,43^ Studies have shown that treatment with broad-spectrum antibiotics increased fecal secretion of steroid hormones and decreased renal excretion, likely due to the lack of deconjugation and subsequently increased loss of conjugated steroid hormones in the feces.^44^ Interestingly, dietary changes such as increased fiber or decreased dietary fat have also been associated with increased fecal excretion and decreased levels of circulating androgenic steroid hormones.^45^ Both an increased dietary fiber and a vegetarian diet low in fat have been shown to be associated with reduced fecal bacteria β-glucuronidase activity, which would result in a decreased reuptake of excreted sex steroid hormones.^46^ A landmark study in 2019 showed that glucuronidated testosterone and dihydrotestosterone are in high concentrations in the small intestine and are efficiently deglucuronidated by gut-associated microbes, leading to fecal concentrations of free androgens higher than serum levels.^47^ This activity also translates to estrogen compounds, as recent work has shown gut microbial glucuronidase activity can lead to increased circulating estrogen activity.^48,49^ Together, this suggests that the gut microbiome is well equipped to deconjugate endogenously excreted sex hormones and that this activity can be altered by host factors such as diet and medications.

In addition to modulating the fecal excretion of conjugated sex steroids, gut-associated microbes have also been shown to harbor gene products for enzymes capable of metabolizing numerous sex steroid hormones, further modulating their effects on the host (Table 1). One of the most studied examples of this is *Comamonas testosteroni*, which was reported in 1956 to inactivate free testosterone.^50^ Later work uncovered a multi-step enzymatic process by which *C. testosteroni* degrades testosterone, an activity which was shared by *Mycobacterium neoaurum*, both constituents of the human gut microbiome.^51^ Cortisol, also found in the lumen of the large intestine after excretion in bile, can be metabolized by DesAB containing microbes such as *Clostridium scindens*, *Butyricicoccus desmolans*, and *Clostridium cadaveris* to form 11β-hydroxyandrostenedione.^52–54^ Previously overlooked for their potential relevance in human physiology or pathophysiology, recent literature surrounding 11-oxygenated androgens has not only been shown them to be potent androgens themselves but has been implicated in numerous disease states including PCOS and prostate cancer, although the focus of most prior work has been on endogenously produced as opposed to byproducts of gut microbial metabolism.^55,56^

**Table 1.** Human gut-associated microbes capable of metabolizing steroid hormones.

Enzymatic activity	Potential androgen metabolism	Gut-associated microbe harboring enzyme	Potential physiologic impact
3α-hydroxysteroid dehydrogenase	Testosterone inactivation	Numerous gut-associated microbes	Feminization via decrease in circulating androgens
3β- hydroxysteroid dehydrogenase	Testosterone, estradiol inactivation	Eggerthella lenta, Mycobacterium neoaureus, Klebsiella aerogenes	Decrease in serum androgens, estrogens
17β- hydroxysteroid dehydrogenase	Testosterone inactivation	Eggerthella lenta, Actinobacteria	Feminization via decrease in circulating androgens
17α- hydroxysteroid dehydrogenase	Epitestosterone generation	Clostridium scindens	Increase in serum androgens
Steroid 17,20 desmolase	Androgen generation from cortisol	Clostridium scindens	Increase in serum androgens
Steroid aldolase	Testosterone inactivation	Mycobacterium tuberculosis	Feminization via decrease in circulating androgens
Beta glucuronidase	Liberation of androgen from β-glucuronide conjugation	Numerous gut-associated microbes	Increase in serum androgens, estrogens

Another class of steroid-metabolizing enzymes possessed by constituents of the gut microbiome are pyridine nucleotide-dependent hydroxysteroid dehydrogenases (HSDH). HSDHs can target numerous hydroxyl groups present on both sex steroids and bile acids.^57^ These enzymes can oxidize and epimerize steroids, changing their configuration, and thereby altering their physiological effects. Numerous *Clostridium* species have been shown to harbor genes expressing HSDHs active on sex hormones, including 17α-HSDH.^54,58^ Numerous species within the Bacteroides genus, another common constituent of the gut microbiome, have demonstrated the ability to metabolize sex hormones via HSDH activity.^53^ *Eggerthella lenta* was determined to preferentially oxidize hydroxyl groups on both bile acids and sex hormones.^59,60^ Additionally, prior work has shown the ability for *Eggerthella lenta* to convert cortisone to 11beta-hydroxy-progesterone from cortisone.^61,62^ Taken together, this suggests a robust metabolic potential possessed by the gut microbiome to metabolize sex hormones. Given the extent of metabolism of steroid molecules by the gut microbiome, there has been a push in recent years to regard this “*sterolbiome*” as its own endocrine organ.^63^

## Alterations to the sterolbiome in cirrhosis may contribute to sexual dysfunction

The effect the gut microbiome has on circulating sex hormone concentrations is an area of increasing interest. A study on changes in gut microbiome profiles in healthy men and women with high versus low levels of circulating sex hormones (testosterone and estradiol, respectively) showed that individuals with higher circulating sex hormone concentrations also had higher diversity of their gut microbiome.^64^ In patients with advanced liver disease, gut microbial diversity decreases as liver disease progresses, suggesting a role decreased gut microbial diversity has in altering circulating sex hormones in cirrhosis. More recent work has shown there are major differences in the gut microbial function between men and women in cirrhosis with HE. The gut microbiota in male patients with cirrhosis have been shown to match that observed in females, with an increase in microbes associated with androgen metabolism.^65^

Even studies of individual constituents of the gut microbiome have supported the idea that gut microbes can impact both circulating sex hormone concentrations and be associated with disease states. Studies of gut-associated microbe *Klebsiella aerogenes* have identified a 3β-HSDH that is capable of degrading estradiol and was found in higher amounts in premenopausal women with depression compared to those without depression.^66^ *Mycobacterium neoaurum* was isolated from fecal samples of testosterone deficient patients, was found to have 3β-HSDH activity against testosterone, and its presence was noted to be associated with depression in males.^67^ Recent work completed by our group showed that men with cirrhosis had higher levels of serum androstenedione due to decreased microbial degradation.^65^ These findings are important in this patient population, as prior work has shown that serum androstenedione is high in men with cirrhosis due to suppression of the hypothalamic-pituitary axis, and that androstenedione is a more efficient metabolic precursor to estrogen than testosterone.^10^ Other work in animal models has shown that oral rifaximin treatment, a mainstay treatment of hepatic encephalopathy (HE) in patients with cirrhosis, led to an increase in testicular volume, decrease in toxic reactive oxygen species in testes, and quantifiable increases in circulating testosterone compared to no rifaximin controls.^68^

Even outside of modulation of circulating sex hormones, there are well-established mechanisms by which the gut microbiome can influence cognition and mental health.^69,70^ Alterations in the composition of the gut microbiome have been noted in patients with a variety of psychiatric disorders, suggesting a role of the gut microbiome in the development of neuropsychiatric pathophysiology.^71,72^ Prior studies have shown that alcohol cravings, anxiety, and depression all positively correlate with increased intestinal permeability found even in the early stages of alcohol use disorder.^73^ Changes to the gut microbiota have also been shown to drive the development of neuroinflammatory responses and brain dysfunction in cirrhosis.^74^ Taken together, these data support an important role the gut microbiome plays in circulating sex hormones and the development of sexual dysfunction in patients with cirrhosis.

## Fecal microbiota transplantation: a potential treatment for sexual dysfunction in cirrhosis

Fecal microbiota transplantation (FMT), initially pioneered for its effective treatment of recurrent *Clostridioides difficile* infections, has also been studied in the setting of gut-liver-brain axis disorders in cirrhosis.^75–78^ FMT involves a drastic alteration of the host gut microbiome, where fecal suspension from a donor is administered to the recipient through either oral, rectal, or endoscopic means. This results in major changes to the composition of the recipient gut microbiome composition, along with its metabolites.

The initial testing of FMT in the treatment of HE was performed in 2016 and showed promising results, with improvement in cognition with FMT that reversed after cessation of FMT.^79^ Subsequent studies including two randomized controlled trials and open-label series in patients with cirrhosis with recurrent HE showed improvement in both cognition and decreased gut microbial dysbiosis after treatment with FMT.^75,79–81^ These data support that FMT in patients with cirrhosis can be successful not only in treating HE but also in treating impaired daily function.

To date, there have been no FMT trials targeting sexual dysfunction in healthy patients or in patients with cirrhosis. However, there is precedence in the literature that such major manipulation of the gut microbiome may lead to improvement in factors leading to sexual dysfunction. Therapeutic manipulations of the gut microbiome through FMT have shown impacts on circulating sex steroid concentrations. Mice induced to have decreased circulating testosterone in the setting of high fat diets were treated with FMT, with improvement in circulating testosterone levels.^82^ A subsequent study supported these results, showing that testosterone levels decreased by systemic infection in the setting of lipopolysaccharide administration was ameliorated by FMT, with improvement of circulating testosterone levels.^83^

This suggests FMT may have a role as a potential therapy in patients who may be poor candidates for other medical therapy. However, further exploration of FMT as a potential treatment option is needed. In designing a potential FMT trial for the treatment of sexual dysfunction in patients with cirrhosis, there are a few factors to consider. Prior work has shown significant differences in the microbiome composition of males and females with cirrhosis, so FMT donors should be sex-matched.^65^ Additionally, numerous comorbid conditions can affect both sexual dysfunction and gut microbiome, such as diabetes and obesity, and should be avoided in potential FMT donors.^84,85^ While prior work has suggested specific taxa may be associated with either increased or decreased concentrations of circulating sex hormones, this work has not been replicated in patients with cirrhosis. Additionally, prior work has shown that increased gut microbial diversity is linked to increased circulating sex hormones.^86^ As liver disease progresses to cirrhosis, more profound changes can occur in the gut microbiome, leading to decreased amounts and diversity of gut-associated microbes.^76^ Therefore, the goal would be, in addition to the usual FDA restrictions for donors, to sex-match recipients with relatively younger donors without sexual dysfunction elicited through questionnaires and sex hormone analysis. It would also be important to monitor circulating sex hormones both before and after FMT to assess response, as well as longitudinally to monitor duration of response. Numerous clinical markers of sexual dysfunction, as reviewed above, could be utilized to monitor improved sexual dysfunction, but most importantly, evaluating patient-reported outcomes focused on this improvement is critical. Given the success in FMT in treating other disorders of the gut-liver-brain axis and how sexual dysfunction fits into this axis, it is reasonable to hypothesize that FMT, which has been shown to be safe in the treatment of other consequences of cirrhosis, may be a tool in our armamentarium to improve sexual dysfunction as well.

## Conclusions and perspectives

The gut-liver-brain axis is an important physiologic crossroads between numerous organ systems that, when disrupted, leads to a myriad of clinical consequences. While the best studied perturbation of the gut-brain-liver axis in cirrhosis is HE, sexual dysfunction is also critical aspect of overall wellbeing. Rates of sexual dysfunction are likely underdiagnosed in patients with cirrhosis. Even when identified, the current treatments for sexual dysfunction are limited in patients with cirrhosis due to the potential for side effects in this complex patient population; therefore, this disorder goes undertreated as well. The initial step in effective treatment of sexual dysfunction in patients with cirrhosis is clinical recognition. Sexual dysfunction is an important aspect of overall patient health and it is important for clinical providers to appropriately screen, particularly in patients that already have clinical signs of disruption of the gut-liver-brain axis such as HE.

It is important to recognize that barriers do exist in the treatment of sexual dysfunction in this complex patient population. Recent studies have shown that sildenafil can be used safely in patients with cirrhosis, however this pharmacologic agent only targets a single aspect of sexual dysfunction in males.^39^ In order to identify safer and more effective avenues for therapeutics, we must better understand the mechanism by which patients with cirrhosis develop sexual dysfunction. There is a growing body of literature supporting the notion that the gut microbiome has a propensity to metabolize sex hormones and may play a role in the homeostasis of sex hormone concentrations in both physiologic and pathophysiological states. Given the significant changes to the composition of the gut microbiome in cirrhosis, these disruptions to the gut-liver-brain axis in cirrhosis present a newly discovered avenue by which these patients develop sexual dysfunction. Numerous bacteria have already been identified that not only carry genes capable of biotransformation of sex hormones but also are altered in specific disease states such as depression. Disruptions to the gut microbiome, such as those that occur in patients with cirrhosis, can alter its ability to metabolize sex hormones and may contribute to the hormonal imbalances that are present in cirrhosis, which drive sexual dysfunction. Further studies are needed to better understand whether changes in the constituents of the gut microbiome correlate to changes in serum sex hormone concentrations as well as clinical evidence of sexual dysfunction. Additionally, advanced therapeutics altering the constituents of the gut microbiome, such as fecal microbiome transplant, may be advantageous therapies to restore balance to the gut-liver-brain axis in these patients given their good safety profile. The goal of further study in this area would be to establish the gut microbiome as a safer therapeutic avenue to address sexual dysfunction compared to currently available therapies in patients with cirrhosis.
